# Modified Management of Abdominal Compartment Syndrome Using the Arrowhead Regional Medical Center Plication Method (ARMC PM): A Bedside Plication Approach Based on the Bogota Bag

**DOI:** 10.7759/cureus.86130

**Published:** 2025-06-16

**Authors:** Aldin Malkoc, Angel Guan, Gunjan Bhat, Harpreet Gill, Amanda Daoud, Payam Falatoonzadeh, David T Wong

**Affiliations:** 1 General Surgery, Arrowhead Regional Medical Center, Colton, USA; 2 Surgery, Arrowhead Regional Medical Center, Colton, USA

**Keywords:** abdominal compartment syndrome, bogota bag, open abdomen surgery, plication, trauma surgery

## Abstract

Background

Several devices are marketed to help with abdominal closure. We aim to describe a novel approach to the management of abdominal compartment syndrome (ACS) using relatively inexpensive and available materials.

Methods

Fifty-two trauma patients were identified with abdomens left open for staged laparotomy. We utilized a method for treatment of ACS that involved suturing a folded transparent drape to the fascial edge using a continuous non-absorbable suture along with a moistened gauze roll that is placed along the edges of the fascia next to a suction drain, creating a vacuum wound dressing covered by Ioban^TM^ (3M Health Care, St. Paul, MN, USA). At the bedside, the outer layer of the drape is plicated with a heavy running suture to tighten the bag and approximate the fascia. The Arrowhead Regional Medical Center Plication Method (ARMC PM) is named after the hospital where it was developed.

Results

Complete fascial and skin closure was achieved in 100% of patients treated for ACS with the ARMC PM technique (p=0.026). Among these patients, the median duration of Bogota bag placement prior to definitive closure was three days. When comparing this cohort to a historical control group in which definitive closure was not achieved, there was a statistically significant reduction in mortality (17% vs. 71%; p=0.048). Notably, no cases of bowel evisceration or enteric fistula occurred with this method.

Conclusion

The ARMC PM is technically simple, allowing for the successful approximation of fascia at the bedside without the need for general anesthesia, thereby minimizing the transport of critically ill patients to the operating table. This technique is an effective and inexpensive method in the management of open abdomens.

## Introduction

Various conditions can lead to or put the patient at risk for abdominal compartment syndrome (ACS), where life-restoring treatment is decompressive laparotomy with delayed abdominal closure [1,2]. ACS leads to end-organ damage, including cardiovascular, renal, hepatic, respiratory, and systemic complications [3]. Various open abdomen techniques exist to help prevent and treat ACS while allowing drainage of abdominal fluids resulting in temperature derangements, and loss of abdominal domain [1].

The treatment and prevention of ACS are crucial for patient outcomes. Various open abdomen techniques exist to help prevent and treat ACS while allowing drainage of abdominal fluids, temperature derangements, and loss of abdominal domain [3]. Several techniques for temporary closure of the abdominal cavity are reported, which include vacuum-assisted closure (VAC), classic Bogota technique, and Barker technique. Additional techniques include the use of commercial devices that aid in the gradual closure of the abdominal wall, such as the AbThera^TM^ and the Wittmann patch^TM^ [4,5]. The VAC system consists of a sterile drape over a reticulated foam placed over the abdominal contents with suction that generates continuous sub-atmospheric pressure. The classic Bogota bag technique consists of a pre-sterilized bag that is sutured to the skin with a negative pressure dressing on top. The Barker technique utilizes an open polyethylene sheet on top of the abdominal viscera, a humid dressing with two suction drains over that sheet, along with an adhesive sheet throughout the wound. The suction drains are used to allow continuous negative pressure similar to a VAC system without direct contact of the abdominal content with a reticulated foam [4]. The AbThera^TM^ is a more recent commercial appliance similar to the Barker technique with the addition of an embedded reticulated foam within the polyurethane layer that allows evacuation of the lateral gutters of the abdomen with a similar VAC system, except the bowel is not directly in contact with the foam [6]. The Wittmann patch^TM^ is a commercial device that allows for serial abdominal closure within an intensive care unit (ICU) setting [5]. Once a patient is considered stable, complete abdominal wall closure is attempted. If complete closure is not possible, surgeons can elect to aid abdominal closure with closing skin only initially, or use an absorbable mesh followed by skin grafting until a subsequent definitive abdominal closure is possible [3,5].

This study describes a novel approach to managing an open abdomen using relatively inexpensive and readily available material from the standard operating room inventory. This novel approach aims to satisfy desirable characteristics for an abdominal closure device, which should include prevention and treatment of ACS with fluid drainage; prevention of evisceration of abdominal contents; control of temperature; assist fascial closure by preventing fascial retraction and lost of abdominal domain; allow visual assessment of bowel viability and fluid content; cost-effectiveness; inert material minimizing inflammation, granulation and adhesions; minimize operative transport by bedside abdominal plication; ability to release pressure if ACS redevelops; and offer simplicity. We compare our method, which allows attachment to fascia, and report on our outcomes.

## Materials and methods

This retrospective study was conducted at Arrowhead Regional Medical Center (ARMC), a level I trauma center and the county hospital for San Bernardino, California, with over 130,000 annual emergency department visits. After obtaining Institutional Review Board (IRB) approval (IRB #22-03), a five-year chart review (2015-2020) was performed to identify trauma patients who underwent open abdominal management with temporary closure. Inclusion criteria consisted of all trauma patients aged 18 years or older whose abdomens were left open during the initial operative management and who survived beyond the first 24 hours. This included patients undergoing decompressive laparotomy for clinically diagnosed ACS, as well as patients undergoing damage control laparotomy (DCL) for physiologic derangement or contamination control where the abdomen was intentionally left open. Exclusion criteria included patients who died within 24 hours of surgery and patients without documentation of abdominal closure technique.

Patients were stratified into two categories based on the indication for temporary abdominal closure (TAC). The first was therapeutic management of ACS, defined as elevated intra-abdominal pressure with evidence of end-organ dysfunction [1], and the second was prophylactic management, defined as use of an open abdomen in the absence of ACS (for DCL or to prevent ACS development in high-risk patients).

The primary comparison was between patients managed with the ARMC Plication Method (ARMC PM), a modified Bogota bag technique involving fascial suturing of a folded sterile plastic sheet, and those managed with other TAC techniques not involving fascial attachment. Data extracted included demographics, comorbidities, Injury Severity Score (ISS), closure technique, days to closure, number of closure attempts, fascial closure success, mechanism of injury, ACS recurrence, hospital length of stay, discharge disposition, success of closure and re-approximation of fascia, and mortality.

Modified Bogota bag plication procedure (ARMC PM)

This technique was developed and refined over two decades ago at ARMC. During the initial exploratory laparotomy, the abdomen is intentionally left open, and a Top-Draper^®^ Warmer drape (ORS-100, O.R. Solutions, Inc., Chantilly, VA) is applied. The drape is folded to create at least four layers and then sutured directly to the fascial edges using a continuous heavy suture, such as 0-looped Polydioxanone II (PDS II®, Ethicon, Inc., Somerville, NJ, USA) or thicker, with large, advancing bites. This creates a transparent, inert barrier that allows for direct visualization of the abdominal contents.

**Figure 1:**
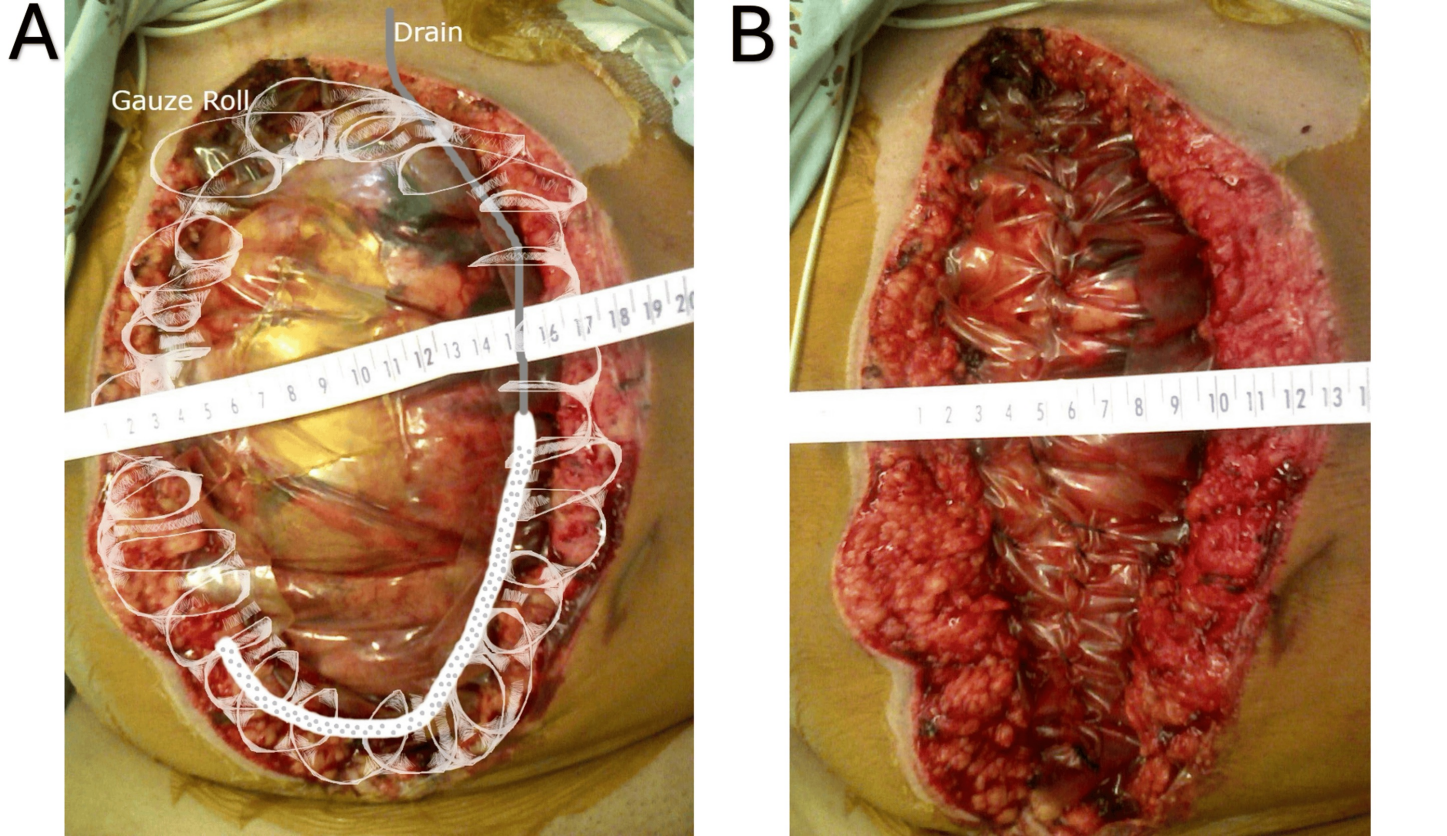
(A) This figure shows a diagram of the placement of surgical drains and gauze rolls atop the sutured inner drape. The ruler across the top indicates how far the abdominal fascia is stretched before the bedside plication. (B) The external plastic drape layer is serially plicated at the bedside over successive dressing changes. The ruler shows how much closer the abdominal fascia has been brought together.

Next, a plain gauze roll with an embedded suction drain is placed along the fascial edges and exposed soft tissue. This acts as a wound vacuum dressing, promoting fluid drainage and protecting the abdominal domain. The entire assembly is then sealed with a 3M® Ioban^TM^ Sterile Antimicrobial Incise Drape (3M Health Care, St. Paul, MN, USA). Suction tubing is positioned along the upper abdomen, and the Ioban^TM^ is carefully wrapped to fully encircle the tubing, preventing air leaks (Figure 1A). Continuous low wall suction is applied (120 mmHg) at the bedside.

Once clinical signs and symptoms of ACS resolve, the Ioban^TM^ drape is removed, and the Top-Draper^®^ Warmer drape is plicated at the bedside. This is performed using the same 0-looped PDS II suture through only the outer layers of the drape, leaving the underlying layer intact to protect the bowel (see Figure 1B). If fluid output remains significant, a fresh Ioban^TM^ layer is reapplied over the site along with suction. This setup continues to facilitate fluid drainage, minimize evaporative heat loss, and allow precise fluid loss monitoring.

**Figure 2:**
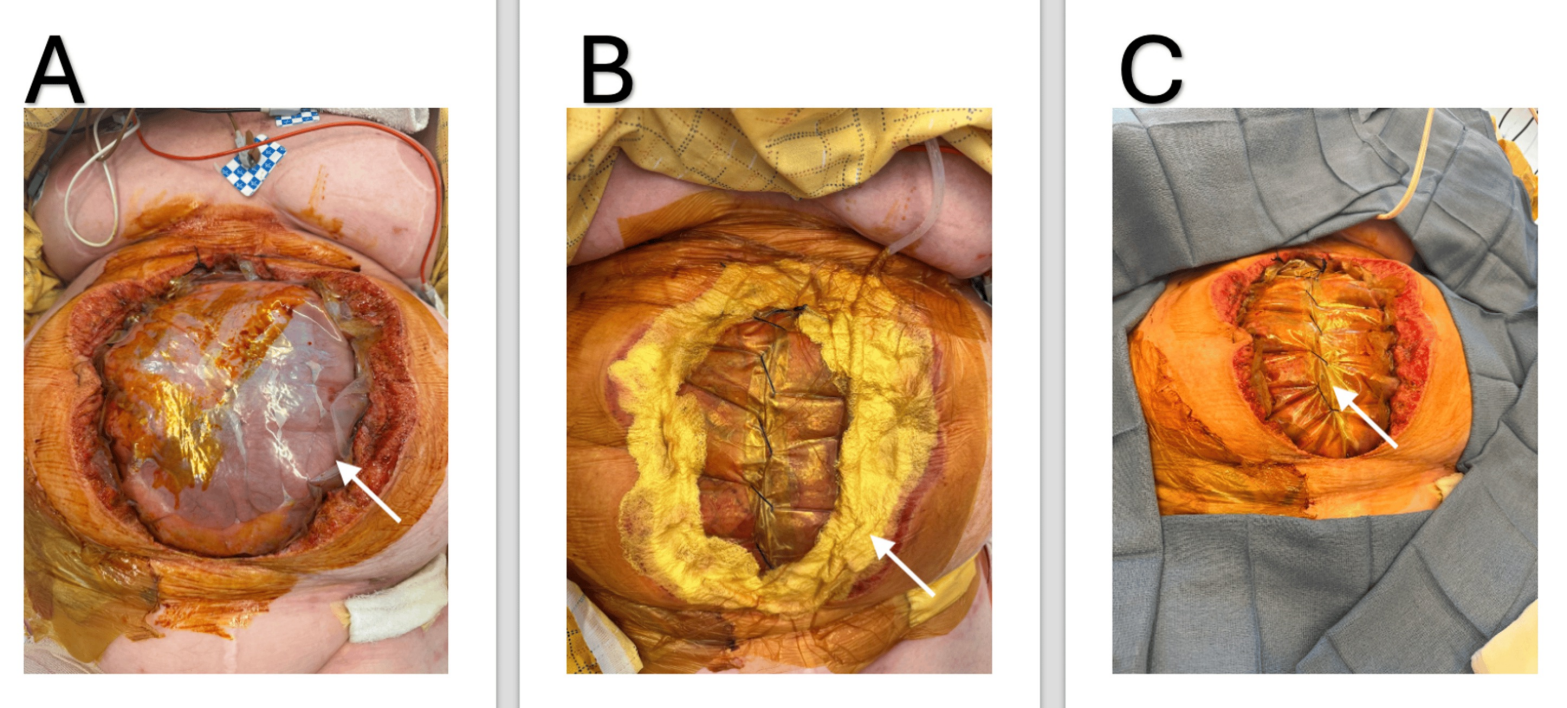
Stepwise application of the ARMC PM technique. A) Inner plastic drape secured directly to the fascial edges with a running suture, creating the foundational barrier layer (white arrow indicating the inner plastic drape). B) Placement of surgical drains and gauze rolls atop the sutured inner drape, followed by coverage of the entire abdomen with an occlusive Ioban^TM^ sheet to ensure negative pressure compatibility and seal integrity (white arrow indicating the inner white gauze dressing). C) External plastic drape layer is serially plicated at the bedside over successive dressing changes, facilitating progressive abdominal domain reduction and tension-free delayed primary closure (white arrow indicating the center plication of the inner plastic drape). ARMC PM: Arrowhead Regional Medical Center Plication Method

Figure 2 shows the progressive process. When the fascial edges approximate to within 4-6 cm, the patient is returned to the operating room for definitive fascial and skin closure, with or without the use of retention sutures, depending on intraoperative tension.

Statistical analysis

Univariate analyses were performed using chi-squared for categorical data. Continuous data that was not normally distributed was analyzed using non-parametric Mann-Whitney U tests. Statistical analysis was then performed using the IBM SPSS Statistics for Windows, Version 27 (Released 2021; IBM Corp., Armonk, New York, United States). Continuous data was represented as median with interquartile range. In this analysis, a p-value less than 0.05 was considered statistically significant.

## Results

Appliance placed for ACS

An inquiry of the ARMC electronic health record over a five-year period yielded 52 discrete trauma patients whose abdomens were left open, with 13 patients treated for ACS. There was no statistically significant difference in age and gender between groups. Complete abdominal closure with fascia (ARMC PM method) versus unsuccessful closure was statistically different (p=0.026). The average length of hospitalization between successful and failed closure was not statistically significant (p=0.086). Glasgow Coma Scale, ISS, and blunt versus penetrating mechanism of injury did not statistically correlate to outcomes (p>0.05). Patients with successful closure had a 17% (N=1) mortality, lower than the 71% (N=5) mortality rate for patients with failed closure (p=0.048). These results are summarized in Table 1.

**Table 1:** Patient characteristics for Bogota bag placement in the treatment of true abdominal compartment syndrome. Continuous data are presented as median with interquartile range (IQR). (a) Analyzed using the non-parametric Mann-Whitney U test. All other comparisons were performed using the Chi-square test. * indicates a statistically significant p-value. A p-value < 0.05 was considered statistically significant. ARMC PM: Arrowhead Regional Medical Center Plication Method

Fascial closure	Success (N=6)	Failure (N=7)	p-value	Test statistic numerical value
Demographics				
Age, year, median (IQR)^a^	49.5 (34.8, 56.3)	47 (40, 64)	0.775	19
Gender, male %	4 (66)	5 (71)	0.853	0.034
Length of hospitalization in days, median (IQR)^a^	21.5 (15, 67.5)	7 (2, 41)	0.086	9
Health status/comorbid disease				
Glasgow Coma Scale, median (IQR)^a^	15 (3, 15)	15 (8, 15)	0.575	18
Injury Severity Score, median(IQR)^a^	31.5 (25, 38.8)	29 (25, 38)	0.885	20.5
Injury type, blunt (%)	5 (83)	6 (86)	0.453	0.014
Mortality (%)	1 (17)	5 (71)	0.048*	3.899
Bogota bag considerations				
Bogota sutured to fascia, ARMC PM (%)	6 (100)	3 (33)	0.026*	4.952

Prophylactic treatment of ACS

Out of the 52 patients studied, 39 patients had the ARMC PM group placed prophylactically. Similar to the ACS group, there was no statistical difference seen when comparing patients with successful versus unsuccessful abdominal closure in regards to age, gender (male), or number of days hospitalized. Mortality in patients who had Bogota bags placed prophylactically with failed fascial closure was 71% (N=5) and was statistically significant, p<0.001, compared to those with 3% (N=1) successful closure after prophylactic placement. No patients in our ARMC PM group had the formation of fistulas. The results are summarized in Table 2.

**Table 2:** Patient characteristics for Bogota bag placement in the prophylactic treatment of abdominal compartment syndrome. Continuous data are presented as median with interquartile range (IQR). (a) Analyzed using the non-parametric Mann-Whitney U test. All other comparisons were performed using the Chi-square test. * indicates a statistically significant p-value. A p-value < 0.05 was considered statistically significant. ARMC PM: Arrowhead Regional Medical Center Plication Method

Fascial closure	Success (N=32)	Failure (N=7)	p-value	Test statistic numerical value
Demographics				
Age, year, median (IQR)^a^	25 (19, 39)	34 (21, 49)	0.332	85.0
Gender, male %	24 (75)	5 (71)	0.845	0.034
Length of hospitalization in days, median (IQR)^a^	16 (8, 28)	1 (1, 46)	0.057	60.0
Health status/comorbid disease				
Glasgow Coma Scale, median (IQR)^a^	14 (7, 15)	12 (3, 14)	0.240	81.0
Injury Severity Score, median (IQR)^a^	26 (17, 35)	33 (19, 41)	0.463	92.0
Injury type, blunt (%)	15 (47)	5 (71)	0.239	1.40
Mortality (%)	1 (3)	5 (71)	<0.001*	20.6
Bogota bag considerations				
Bogota sutured to fascia, ARMC PM (%)	17 (53)	2 (29)	0.239	1.40

## Discussion

There have been various studies that have discussed the risks and benefits of various techniques in the closure of an open abdomen. Classically, the Bogota bag came from a large sterile volume resuscitation bag and was sutured to the skin [6]. The advantages of using the Bogota bag method allowed for DCL and ACS at a low cost without specialized training. The largest cost saving is from reducing repeated operating room usages when plicating the fascial edges at the bedside. However, there are downsides to using the Bogota bag when suturing it to the skin, such as fascial retraction with loss of abdominal domain and evisceration with skin erosion and necrosis [7].

Another technique uses a VAC system, which consists of a plastic drape over a reticulated foam or gauze placed over the abdominal contents with suction that generates continuous sub-atmospheric pressure. The classic Bogota bag technique consists of a pre-sterilized bag sutured to skin with negative pressure and gauze on top. The Barker technique utilizes an open polyethylene sheet on top of the abdominal viscera, a humid gauze dressing with two suction drains over that sheet, and an adhesive sheet throughout the wound. The suction drains allow continuous negative pressure and fluid evacuation similar to a VAC system, without direct contact of the abdominal content with reticulated foam [4]. The Apthera^TM^ is a commercial appliance similar to the Barker technique with an embedded reticulated foam within the polyurethane layer that allows evacuation of the lateral gutters of the abdomen. This system is a similar VAC system, except the bowel is not directly in contact with the foam [6]. The Wittmann patch^TM^ is another commercial device that allows for serial abdominal wall approximation at the bedside, secured onto the fascia on top of abdominal contents [5]. The ARMC PM technique differs from the Wittmann patch^TM^ as it showcases differences in the materials used, resource availability, and feasibility in non-operating room settings.

Following these temporizing techniques, final abdominal closure is attempted in the operating room. If complete fascial closure is impossible, skin-only closure or absorbable mesh closure with skin grafting is performed, accepting a large ventral hernia [1,5].

While studies have found leaving abdomens open is a useful technique for treating ACS and DCL, poor closure rates and frequent trips to the operating room prompted us to develop a modification to existing methods [6,7]. Over 20 years, the method was modified to incorporate these features: prevention and treatment of ACS with accurate measurement of fluid drainage by using a negative pressure wound dressing; prevention of inflammation and evisceration of abdominal contents by suturing an inert plastic sheet to the fascia, minimizing granulation and adhesions and theoretically decreasing small bowel obstruction and entero-atmospheric fistulas; control of temperature with a closed wound system limiting evaporative heat loss; facilitate successful fascial closure by preventing fascial retraction and loss of abdominal domain; allow visual assessment of bowel viability and fluid content via the transparent sheet with vacuum dressings placed only on the skin edges; use of cost effective material; allow for bedside plication with protection of underlying abdominal content to minimize unnecessary transport and operative time; reversible release of plication at bedside if abdominal pressures increase in the post operative period; and simplicity in management. In our five-year study, we determined that the ARMC method with facial attachment compared to sutured/stapled to skin was more effective in delayed primary closure and prevention of bowel evisceration.

Improvement in primary fascial closure is facilitated by prevention of fascial retraction and loss of domain. This was shown in a multicenter prospective trial using mesh-mediated fascial traction and VAC [8,9]. Complete fascial closure was successful in six of nine patients treated for ACS, where the abdominal sheet was sutured to the fascia. Among the three ACS-treated patients who had failed closure, two died of multi-system failure, and one, who had a penetrating injury, developed sepsis and required multiple surgeries. These failures are more indicative of the severity of the underlying injuries rather than the management of an open abdomen. All ACS-treated patients with blunt injuries who survived achieved successful delayed primary closure, as detailed in Table 1.

The ARMC PM facilitated abdominal closure within a median of three days, effectively preventing bowel evisceration or fistula formation. The transparent drape allowed daily assessment of bowel viability and identification of fluid sources, such as bleeding, bile/succus leak, or ascites. The method also minimized evaporative and thermal losses while enabling accurate measurement of the evacuated fluid. The technique’s use of multiple folded sheets allowed for abdominal plications at the bedside without the need for general anesthesia, thereby reducing the risk of bowel injury. During suture plication, the superficial sheets can be pinched away from the lower layer adjacent to the bowel, ensuring that the abdominal contents are never exposed or close to the suturing. This approach significantly reduced the risk of contamination, inflammation, and the eventual development of a frozen abdomen, classified as Bjorck grade III [10].

Since the plastic drape is still intact during or after plication, the plication can be released at any time by cutting the continuous suture, thus returning the plastic drape to its expanded form. Only one patient required release of the plication due to the re-development of ACS. Ioban^TM^ film and drain were then reapplied without returning to the operating room.

Several devices are marketed to help with abdominal closure. Most of the techniques described require the patient with an open abdomen to return to the operating room in a sterile environment for adjustments and attempted closures. This study demonstrates that the ARMC PM, with attachment to fascia and plication, is a safe and effective temporizing approach for open abdomens. This study found a higher incidence of successful abdominal closure with the ARMC PM compared to techniques that do not utilize the fascia for patients with true ACS. However, there was no statistical difference found between the groups when performed prophylactically, which is logical since the fascia is less likely to retract without ACS.

This study had several limitations. As a retrospective review from a single center, the total number of patients is low and potentially subject to type I error. This is because DCL requiring an open abdomen for management is seen in the most severely injured, as seen in the average ISS score >25. The smaller study also does not allow us to separate comparisons to specific techniques. Since the surgeons only used either the ARMC PM or techniques that avoided the fascia with either the modified Barker technique or AbThera^TM^, other techniques, which utilize the fascia, cannot be compared. Because the technique chosen is not randomized, bias is introduced with surgeon preference on the type of technique and when an abdomen is left open prophylactically. To a certain extent, this bias is minimized by the separation of the groups, but it further lowers our power in the study.

Additionally, the significant mortality benefits in successfully closed abdomens should be interpreted with caution and cannot be solely attributed to the ARMC PM. This study found that successful fascial closure is associated with a higher survival rate for both groups (Tables 1-2). As a retrospective study, it is not designed to demonstrate cause and effect. However, it posits that successful closure with less fluid loss, reduced inflammation, and protection of hollow viscus injury from fistula formation can improve outcomes [8,9]. The inability of fascial closure may be a reflection of unresolved underlying medical or surgical issues, which makes failure more of a prognostic indicator rather than a cause of mortality. However, the ISS and injury type were not statistically different, supporting the argument that failure of facial closure makes a significant contribution to mortality. Future research should include larger databases and involve a multicenter trial to achieve significant power to directly compare specific techniques as well as determine specific mortality-reducing benefits.

## Conclusions

This study may offer a useful approach to a modified technique for managing open abdomens using inexpensive and readily available material from either standard operating room inventories or ICU inventories. The technique is technically simple and may allow for more successful approximation of fascia at the bedside without general anesthesia, minimizing the transport of critically ill patients to the operating table. The ARMC PM attempts to address some of the concerns of other methods, including fascial retraction, bowel protection, and inflammation. Based on our experience in this study, this technique shows potential utility in the management of open abdomens.
